# Effect of short-term oral nutrients after hospital discharge on postoperative muscle loss and survival in gastric cancer patients

**DOI:** 10.3389/fonc.2025.1697609

**Published:** 2026-01-06

**Authors:** Yangbin Lu, Jiaqi Yang, Zhixuan Jiang, Jianqiang Huang, Xian Shen, Yuwen Lu

**Affiliations:** 1 Department of Gastrointestinal Surgery, The Second Affiliated Hospital, Wenzhou Medical University, Wenzhou, China; 2 Department of Emergency Surgery, Wenzhou Central Hospital, Wenzhou, China; 3 Department of Gastrointestinal Surgery, The First Affiliated Hospital, Wenzhou Medical University, Wenzhou, China; 4 Department of Dermatology, The Second Affiliated Hospital, Wenzhou Medical University, Wenzhou, China

**Keywords:** skeletal muscle mass, gastric cancer, oral nutrients, survival rate, sarcopenia

## Abstract

**Aim:**

A decrease in skeletal muscle mass in patients with gastric cancer following surgery is closely related to tumor recurrence and poor prognosis. Postoperative enteral nutrition support can accelerate the postoperative recovery of patients with gastric cancer. We aimed to analyze the effectiveness of short-term oral nutritional supplements(ONS) in attenuating postoperative skeletal muscle mass loss in gastric cancer patients after hospital discharge and the impact on long-term prognosis.

**Methods:**

This study included patients who underwent radical gastrectomy at our center between 2014 and 2019. Univariate and multivariate logistic regression analyses were used to analyze the effectiveness of short-term oral nutrition for reducing postoperative skeletal muscle mass loss in patients with gastric cancer. Using cox regression to analyze the effect of ONS and each covariate on overall survival (OS).

**Results:**

ONS attenuated postoperative skeletal muscle loss, and it can improve the prognosis of patients, especially the elderly, those with poor nutritional status, anemia, and patients who have undergone total gastrectomy.

**Conclusions:**

Short-term oral nutrition is an independent protective factor for preventing skeletal muscle mass loss in patients with gastric cancer after surgery. Oral nutrition after surgery can reduce short-term muscle mass loss and is helpful in lowering the risk of death for patients.

## 1 Introduction

Gastric cancer ranks fifth in terms of cancer incidence worldwide and stands as the fourth leading cause of cancer-related fatalities. It is a global disease with a high incidence and mortality, most of them in Asia, Eastern Europe, and South America (1). Although the incidence of gastric cancer is decreasing in most countries, it is expected to increase with the aging of the global population. As global aging becomes increasingly serious, the number of cases of gastric cancer in the elderly will increase, placing a burden on the economy, as well as societies and families. Gastric cancer is currently treated mainly through surgical procedures. Gastric cancer surgery, a digestive tract operation, tends to cause malnutrition and reduced skeletal muscle mass in patients, due to a lack of appetite, difficulty eating (2, 3)and insufficient energy intake. Additionally, postoperative hormonal and metabolic changes can accelerate protein catabolism, resulting in reduced skeletal muscle mass (4). Fukuda et al. found that preoperative sarcopenia was a risk factor for serious postoperative complications in elderly patients with gastric cancer who underwent gastrectomy (5). A large survey found that postoperative skeletal muscle mass loss was associated with a low survival rate after gastric cancer (6, 7).

Patients with gastric cancer are prone to postoperative loss of skeletal muscle mass. Several studies have shown that malnutrition and loss of skeletal muscle mass are associated with a decreased quality of life after surgery (8)and are independent risk factors for recurrence and poor long-term survival prognosis in patients with gastric cancer (9, 10).

Postoperative nutritional management is important for postoperative physical loss reduction, short-term weight loss control and prognosis for survival in patients with gastric cancer. Previous related studies have mostly focused on body weight factors, which are easily affected by factors such as fluid loss or edema. They, therefore, failed to intuitively assess the impact of postoperative oral nutrition on the physical recovery and long-term prognosis of patients. Other previous studies investigated the relationship between oral nutrient deficiency and postoperative skeletal muscle index (SMI) reduction in patients with gastric cancer (11). We, therefore, aimed to explore the relationship between the two and to determine whether postoperative oral nutrient supplements can effectively reduce postoperative skeletal muscle mass loss in patients with gastric cancer, at the Computed Tomography (CT) level. And whether it can improve the prognosis of patients with gastric cancer.

## 2 Methods

### 2.1 Patients

We recruited 850 subjects who underwent radical gastrectomy at our center (Department of Gastrointestinal Surgery, The First Affiliated Hospital, Wenzhou Medical University, Wenzhou, China.) between July 2014 and March 2019. The exclusion criteria were: (1) preoperative neoadjuvant therapy; (2) early gastric cancer; (3) inability to tolerate or refusal to undergo skeletal muscle strength and fitness tests; (4) lack of preoperative or postoperative abdominal CT images. They all took a month’s worth of nutritional supplements, including Enteral Nutritional Suspension(Approval number: H20010284.Each 500ml contains energy 2100KJ (500kcal), protein 20.0g, carbohydrates 61.5g, fat 19.45g.) or Intacted Protein Enteral Nutrition Powder(Approval number:H20030467. Every 100 mL of liquid is made by mixing 21.5 g of powder. Each 500ml contains 2085.5 KJ (496.65 kcal) of energy, 19.89g of protein, 60.63g of carbohydrates, and 19.57g of fat.). Each patient consumed 500ml of nutritional supplements daily. Experienced physicians obtained postoperative survival outcomes via telephone or outpatient visits. Patients underwent a follow-up CT scan three months after discharge, followed by telephone follow-ups every three months. The follow-up endpoint is defined as five years of follow-up or patient death. All subjects underwent open or laparoscopic radical gastrectomy, and all operations were performed by the same surgical team. Anastomotic methods were selected according to the experience of surgeons and the conditions of patients during the operation. Gastric cancer was confirmed by postoperative pathological analysis. The study was approved by the ethics committee of the First Affiliated Hospital of Wenzhou Medical University (No. KY2023-R006).

### 2.2 Data collection

The collected clinical data were categorized into three groups: (1) preoperative basic clinical data included sex, age, body mass index (BMI), preoperative weight loss, abdominal CT images 1 month prior to surgery, grip strength (GS), walking speed, serum albumin concentration (<35 g/L was defined as hypoalbuminemia), serum hemoglobin concentration (<120 g/L for males and <110 g/L for females was defined as anemia), NLR(Neutrophil to Lymphocyte Ratio, Monocyte/lymphocyte),and Charlson Comorbidity Index(CCI); (2) Surgical information and pathological tumor data included surgical method, resection type (distal gastrectomy (DG) or total gastrectomy (TG)), anastomotic method, operation time, tumor length and diameter, tumor differentiation degree, postoperative chemotherapy and Tumor Node Metastasis (TNM) stage. (3) Postoperative clinical data included abdominal CT images 3 months following surgery and Short-term postoperative complications (We evaluated postoperative complications that occurred within 30 days after gastrectomy and graded the complications according to the Clavien system (12), including those of Grade II or higher). All clinical data were collected by dedicated researchers who were unaware of each other and maintained patient confidentiality.

### 2.3 Definition of skeletal muscle mass loss

Preoperative and postoperative abdominal CT images were analyzed using the artificial intelligence (AI)-based muscle segmentation evaluation model independently developed by our center. This project uses convolutional neural networks that rely on a large number of manually sketched training samples to build a fully automated body composition segmentation system at the L3 level). Manually sketched training samples are derived from previous research (13). PreSMI and PostSMI were obtained. Postoperative skeletal muscle mass loss was defined as a skeletal muscle index reduction rate of ≥5%. This index was calculated using the following formula: Skeletal muscle index reduction rate = (PreSmi-PostSMI)/PreSMI × 100%.

### 2.4 Diagnosis of sarcopenia

The diagnostic criteria for sarcopenia are low grip strength and/or slow gait speed(GS) combined with muscle mass loss. The measurement position of GS was with the patient’s elbow 180° in the standing position, slowly squeezing an electronic hand dynamometer (EH101: Zhongshan Camiry Electronics Co., Ltd., China) with the dominant hand. The maximum value of three repeated tests was recorded, with each interval lasting 15 min. The threshold value for males was 26 kg, and for females it was 18 kg. Each patient also underwent a 6 m step speed test. Similarly, the maximum value of three repeated tests was recorded using a manual stopwatch, with a threshold value of 0.8 m/s.

### 2.5 Data analysis

The primary endpoint was whether skeletal muscle mass loss occurred 3 months following gastric cancer surgery. Univariate and multivariate logistic regression analyses were used to analyze whether oral nutrients were able to reduce postoperative skeletal muscle mass loss in patients with gastric cancer. The covariables included sex, age, BMI, CCI, anemia, hypoalbuminemia, sarcopenia, surgical method, surgical resection type, anastomosis method, TNM stage. We used BMI as a continuous variable, and the linear hypothesis of the continuous variable logit was evaluated using the Box-Tidwell test. Multicollinearity was evaluated using a multiple regression model that examined variance inflation factors. The effect of oral nutritional Supplements (ONS) as well as covariates on OS was analyzed using cox regression, and statistically significant factors(P<0.05) from univariate analyses were included in multivariate analyses in order to identify independent risk factors affecting patients’ OS. Kaplan-Meier curves and log-rank tests were used to determine survival differences between groups. After applying propensity score matching to reduce differences between the two groups, we reanalyzed the impact of ONS on reducing sarcopenia and improving survival rates. p < 0.05 was considered statistically significant. All statistical analyses were performed using IBM SPSS Statistics software (version 25.0; IBM Corp., Armonk, NY, USA).

## 3 Results

### 3.1 Baseline data

**Table 1** shows that we enrolled 850 eliglible participants. Of the total participants, 500 used oral nutrient supplements after discharge from the hospital, and 350 consumed ordinary diets. For the population characteristics, the ONS group was more likely to be anemia (P = 0.049), preferred open surgery(P<0.001), had fewer patients with sarcopenia(P<0.001).

**Table 1:** Patient baseline characteristics.

Factors	Normal diet+ONS	Normal diet	P
	N=500	N=350	
Sex			0.367
Male	353 (70.6%)	257 (73.4%)	
Female	147 (29.4%)	93 (26.5%)	
Age			0.248
≤65	253 (50.6%)	187 (53.4%)	
>65	247 (49.4%)	163 (46.6%)	
BMI	22.8±3.2	22.4±2.9	0.378
CCI			0.139
≤1	411 (82.1%)	301 (86.0%)	
≥2	89 (17.2%)	49 (14.0%)	
Hypoalbuminemia			0.319
0	389 (77.8%)	262 (74.8%)	
1	111 (22.2%)	88 (25.1%)	
Anaemia			0.049
0	337 (67.4%)	213 (60.8%)	
1	107 (32.6%)	137 (39.2%)	
Operation mode			<0.001
open	276 (55.2%)	264 (75.4%)	
laparoscopy	224 (44.8%)	86 (24.5%)	
Type of resection			0.631
distal gastrectomy	331 (62.2%)	212 (60.5%)	
total gastrectomy	189 (37.8%)	138 (39.5%)	
Combined resection	31 (6.2%)	32 (9.1%)	0.107
TNM			0.069
I	187 (37.4%)	105 (30%)	
II	107 (21.4%)	78 (22.3%)	
III	206 (41.2%)	167 (47.7%)	
Sarcopenia	61 (12.2%)	78 (22.2%)	<0.001
Muscle loss	230 (46.0%)	201 (57.4%)	0.01
Complications	125 (25.0%)	99 (28.3%)	0.285
NLR	250 (50.0%)	175 (50%)	1.000
Chemotherapy	266 (53.2%)	201 (57.4%)	0.223

Statistically significant (P<0.05). CCI, Charlson Comorbidity Index; TNM, Tumor Node Metastasis; BMI, body mass index; ONS, oral nutritional supplements; NLR, Neutrophil to Lymphocyte Ratio.

### 3.2 Univariate and multivariate logistic regression analysis

**Table 2** shows that in univariate analysis, BMI, CCI score, anemia, hypoalbuminemia, surgical procedure, sarcopenia, chemotherapy, NLR and postoperative nutritional supplements were significantly associated with postoperative skeletal muscle loss in patients, and after incorporating each covariate(P<0.05) into the multivariate analysis, CCI score(OR = 1.666,P=0.011), anemia(OR = 1.428,P=0.041), chemotherapy(OR = 1.511,P=0.004) and sarcopenia(OR = 1.707,P=0.014) were still significantly associated, and were risk factors for postoperative muscle loss in patients with gastric cancer, ONS(OR = 0.710,P=0.022) and BMI(OR = 0.950,P=0.04) were the Protective factors

**Table 2:** Univariate and multivariate analyses related to muscle loss.

Factors	Univariate analysis	P	Multivariate analysis	P
	OR (95%CI)		OR (95%CI)	
Sex				
Female	Ref			
Male	0.787 (0.583-1.062)	0.117		
Age				
≤65	Ref			
>65	1.040 (0.794-1.361)			
BMI	0.909 (0.869-0.952)	<0.001	0.949 (0.904-0.997)	0.039
CCI				
≤1	Ref		Ref	
≥2	1.572 (1.087-2.275)	0.016	1.626 (1.100-2.402)	0.015
Hypoalbuminemia				
0	Ref		Ref	
1	2.013 (1.451-2.794)	<0.001	1.429 (0.976-2.092)	0.067
Anaemia				
0	Ref		Ref	
1	1.825 (1.372-2.429)	<0.001	1.411 (1.010-1.970)	0.043
Operation mode				
open	Ref		Ref	
laparoscopy	0.719 (0.543-0.952)	0.021	0.845 (0.628-1.141)	0.273
Type of resection				
distal gastrectomy	Ref			
total gastrectomy	0.791 (0.599-1.043)	0.096		
Combined resection				
0	Ref			
1	1.234 (0.737-2.068)	0.424		
TNM				
I	Ref			
II	0.929 (0.639-1.335)	0.673		
III	0.949 (0.698-1.289)	0.737		
Sarcopenia				
0	Ref		Ref	
1	2.410 (1.637-3.547)	<0.001	1.752 (1.149-2.671)	0.009
ONS				
0	Ref		Ref	
1	0.631 (0.479-0.832)	0.001	0.725 (0.543-0.970	0.03

Statistically significant (P<0.05). CCI, Charlson Comorbidity Index; TNM, Tumor Node Metastasis; BMI, body mass index; ONS, oral nutritional supplements; NLR, Neutrophil to Lymphocyte Ratio.

### 3.3 Univariate and multivariate analyses related to overall survival

**Table 3** shows that age, BMI, anemia, hypoalbuminemia, surgical procedure, type of resection, combined organ resection, TNM stage, sarcopenia complications, NLR and postoperative nutritional supplements were significantly associated with patient survival in univariate analyses, and after including covariates with P<0.05 in univariate analysis into multivariate analysis, multifactorial analyses showed that age, (HR = 1.694,P<0.001) type of resection(HR = 1.460,P=0.002), and TNM stage(II/I HR = 2.091,P =0.003; III/I HR=6.493,P < 0.001) were still significantly associated with being independent risk factors for postoperative OS in gastric cancer patients.

**Table 3:** Univariate and multivariate analyses related to overall survival.

Factors	Univariate analysis	P	Multivariate analysis	P
	HR (95%CI)		HR (95%CI)	
Sex				
Female	Ref			
Male	1.151 (0.879-1.508)	0.307		
Age				
≤65	Ref			
>65	1.791 (1.407-2.281)	<0.001	1.694 (1.309-2.193)	<0.001
BMI	0.961 (0.924-1.000)	0.049	0.982 (0.944-1.022)	0.366
CCI				
≤1	Ref			
≥2	0.982 (0.711-1.356)	0.913		
Hypoalbuminemia				
No	Ref			
Yes	1.866 (1.451-2.399)	<0.001	1.274 (0.949-1.711)	0.107
Anaemia				
No	Ref			
Yes	1.630 (1.285-2.068)	<0.001	0.924 (0.695-1.230)	0.589
Operation mode				
open	Ref			
laparoscopy	0.546 (0.415-0.719)	<0.001	0.841 (0.632-1.117)	0.232
Type of resection				
distal gastrectomy	Ref			
total gastrectomy	2.023 (1.596-2.564)	<0.001	1.460 (1.145-1.865)	0.002
Combined resection				
No	Ref			
Yes	1.896 (1.304-2.755)	0.001	1.262 (0.858 -1.858)	0.238
TNM				
I	Ref			
II	2.468 (1.537-3.961)	<0.001	2.091 (1.292-3.384)	0.003
III	7.635 (5.171-11.273)	<0.001	6.493 (4.339-9.716)	<0.001
Sarcopenia				
No	Ref			
Yes	1.822 (1.381-2.403)	<0.001	1.167 (0.854-1.595)	0.332
ONS				
No	Ref			
Yes	0.735 (0.579-0.932)	0.011	0.804 (0.628-1.030)	0.084
Muscle loss				
No	Ref			
Yes	0.933 (0.737-1.182)	0.566		
complications				
No	Ref			
Yes	1.1487 (1.154-1.916)	0.002	1.169 (0.897-1.523)	0.248
NLR				
low	Ref			
high	1.285 (1.013-1.629)	0.039	0.891 (0.693-1.148)	0.370
Chemotherapy				
No	Ref			
Yes	0.796 (0.625-1.014)	0.065		

Statistically significant (P<0.05). CCI, Charlson Comorbidity Index; TNM, Tumor Node Metastasis; BMI, body mass index; ONS, oral nutritional supplements; NLR, Neutrophil to Lymphocyte Ratio.

### 3.4 Subgroup analysis

**Table 4** shows that subgroup analyses suggest that short-term postoperative ONS is more beneficial for OS in the older(HR = 0.619,P=0.002), anemia (HR = 0.667,P=0.026), total gastrectomy(HR = 0.695,P=0.32), and TNM I-phase (HR = 0.352,P=0.008) sarcopenia(HR = 0.558,P=0.026) subgroups of the population.

**Table 4:** Subgroup analyses.

Factors	Univariate analysis	P
	HR (95%CI)	
Age		
≤65	0.850 (0.583-1.239)	0.398
>65	0.619 (0.456-0.841)	0.002
Hypoalbuminemia		
0	0.750 (0.560-1.004)	0.053
1	1.931 (1-1.165)	0.165
Anaemia		
0	0.832 (0.603-1.148)	0.265
1	0.667 (0.466-0.953)	0.026
Type of resection		
distal gastrectomy	0.783 (0.556-1.103)	0.162
total gastrectomy	0.695 (0.499-0.969)	0.032
TNM		
I	0.352 (0.162-0.763)	0.008
II	0.939 (0.510-1.730)	0.840
III	0.882 (0.669-1.163)	0.375
Sarcopenia		
0	0.854 (0.648-1.124)	0.260
1	0.558 (0.334-0.931)	0.026

Statistically significant (P<0.05). CCI, Charlson Comorbidity Index; TNM, Tumor Node Metastasis; BMI, body mass index; ONS, oral nutritional supplementation.

### 3.5 Baseline data after PSM

Age, anemia, hypoalbuminemia, sarcopenia, type of resection, and TNM staging were selected as matching criteria(1:1 matching, match tolerance of 0.02). After matching, both groups comprised 343 patients each, with no statistically significant differences in clinical baseline characteristics between the two groups in **Table 5**. After propensity score matching, open surgery was more common in the normal diet group (P<0.001).

**Table 5:** Patient baseline characteristics after PSM.

Factors	Normal diet+ONS	Normal diet	P
	N=343	N=343	
Sex			0.733
Male	246 (71.8%)	250 (72.8%)	
Female	97 (28.2%)	93 (27.2%)	
Age			0.878
≤65	181 (52.7%)	183 (53.3%)	
>65	162 (47.3%)	160 (46.6%)	
BMI	22.7±3.2	22.5±2.9	0.499
CCI			0.744
≤1	51 (14.9%)	48 (13.9%)	
≥2	292 (85.1%)	295 (86.1%)	
Hypoalbuminemia			0.792
No	255 (74.3%)	258 (75.2%)	
Yes	88 (25.6%)	85 (24.8%)	
Anaemia			0.435
No	202 (58.8%)	212 (61.8%)	
Yes	141 (41.2%)	131 (38.1%)	
Operation mode			<0.001
open	188 (54.8%)	257 (74.9)%	
laparoscopy	155 (45.2%)	86 (25.1%)	
Type of resection			0.141
distal gastrectomy	191 (55.6%)	210 (61.2%)	
total gastrectomy	152 (44.4%)	133 (38.8%)	
Combined resection	21 (6.1%)	31 (9.0%)	0.149
TNM			0.267
I	123 (35.8%)	104 (30.3%)	
II	74 (21.6%)	75 (21.8%)	
III	146 (42.6%)	164 (47.9%)	
Sarcopenia	60 (17.4%)	71 (20.6%)	0.285
Muscle loss	150 (43.7%)	194 (56.5%)	0.001
Complications	88 (25.7%)	97 (28.3%)	0.439
NLR	177 (51.6%)	168 (49.0%)	0.492
Chemotherapy	188 (54.8%)	195 (56.9%)	0.590

Statistically significant (P<0.05). CCI, Charlson Comorbidity Index; TNM, Tumor Node Metastasis; BMI, body mass index; ONS, oral nutritional supplements; NLR, Neutrophil to Lymphocyte Ratio.

### 3.6 Univariate and multivariate logistic regression analysis after PSM

**Table 6** shows that in univariate analysis, BMI, anemia, hypoalbuminemia, surgical procedure, sarcopenia, chemotherapy and postoperative nutritional supplements were significantly associated with postoperative skeletal muscle loss in patients, and after incorporating each covariate into the multivariate analysis, ONS(OR = 0.595,P=0.001),total gastrectomy(OR = 0.637,P=0.006)and BMI(OR = 0.946,P=0.048) was the Protective factors. Sarcopenia(OR = 1.792,P=0.008) and chemotherapy I(OR = 1.413,P=0.033) were risk factors for postoperative muscle loss.

**Table 6:** Univariate and multivariate analyses related to muscle loss after PSM.

Factors	Univariate analysis	P	Multivariate analysis	P
	OR (95%CI)		OR (95%CI)	
Sex				
Female	Ref			
Male	0.798 (0.571-1.116)	0.188		
Age				
≤65	Ref			
>65	1.086 (0.805-1.466)	0.589		
BMI	0.906 (0.861-0.954)	<0.001	0.946 (0.895-1.000)	0.048
CCI				
≤1	Ref			
≥2	1.509 (0.980-2.323)	0.061		
Hypoalbuminemia				
No	Ref			
Yes	1.888 (1.327-2.686)	<0.001	1.410 (0.933-2.133)	0.103
Anaemia				
No	Ref			
Yes	1.742 (1.278-2.373)	<0.001	1.465 (1.023 -2.096)	0.037
Operation mode				
open	Ref			
laparoscopy	0.777 (0.567-1.064)	0.115		
Type of resection				
distal gastrectomy	Ref			
total gastrectomy	0.649 (0.478-0.881)	0.006	0.637 (0.462-0.879)	0.006
Combined resection				
No	Ref			
Yes	1.080 (0.613-1.902)	0.790		
TNM				
I	Ref			
II	1.042 (0.689-1.577)	0.844		
III	0.833 (0.591-1.174)	0.296		
Sarcopenia				
No	Ref			
Yes	2.293 (1.538-3.417)	<0.001	1.792 (1.161-2.765)	0.008
ONS				
No	Ref			
Yes	0.597 (0.441-0.807)	0.001	0.595 (0.435-0.814)	0.001
complications				
No	Ref			
Yes	0.977 (0.698-1.370)	0.895		
NLR				
low	Ref			
high	1.263 (0.936-1.705)	0.127		
Chemotherapy				
No	Ref			
Yes	1.465 (1.081-1.981)	0.014	1.413 (1.028-1.942)	0.033

Statistically significant (P<0.05). CCI, Charlson Comorbidity Index; TNM, Tumor Node Metastasis; BMI, body mass index; ONS, oral nutritional supplements; NLR, Neutrophil to Lymphocyte Ratio.

### 3.7 Univariate and multivariate analyses related to overall survival after PSM

**Table 7** shows that age, anemia, hypoalbuminemia, surgical procedure, type of resection, combined organ resection, TNM stage, sarcopenia, complications, NLR and ONS were significantly associated with patient survival in univariate analyses, and after adjusting for covariates, multifactorial analyses showed that age (HR = 1.666,P=0.001) type of resection(HR = 1.506,P=0.003), ONS(HR=0.751,P=0.038),hypoalbuminemia(HR=1.375,P=0.046)and TNM stage(II/I HR = 1.847,P =0.024; III/I HR = 5.585,P < 0.001) were still significantly associated with being independent risk factors for postoperative OS in gastric cancer patients.

**Table 7:** Univariate and multivariate analyses related to survival after PSM.

Factors	Univariate analysis	P	Multivariate analysis	P
	HR (95%CI)		HR (95%CI)	
Sex				
Female	Ref			
Male	1.140 (0.848-1.532)	0.385		
Age				
≤65	Ref			
>65	2.202 (1.687-2.873)	<0.001	1.666 (1.250-2.220)	0.001
BMI	0.978 (0.936-1.021)	0.308		
CCI				
≤1	Ref			
≥2	0.745 (0.530-1.047)	0.09		
Hypoalbuminemia				
No	Ref			
Yes	2.061 (1.578-2.692)	<0.001	1.375 (1.006-1.879)	0.046
Anaemia				
No	Ref			
Yes	1.730 (1.337-2.240)	<0.001	0.988 (0.725-1.347)	0.940
Operation mode				
open	Ref			
laparoscopy	0.514 (0.378-0.701)	<0.001	0.806 (0.587-1.108)	0.184
Type of resection				
distal gastrectomy	Ref			
total gastrectomy	2.022 (1.560-2.622)	<0.001	1.506 (1.152-1.968)	0.003
Combined resection				
No	Ref			
Yes	2.095 (1.411-3.111)	<0.001	1.364 (0.903-2.060)	0.141
TNM				
I	Ref			
II	2.345 (1.391-3.955)	0.001	1.847 (1.083-3.151)	0.024
III	7.366 (4.803-11.298)	<0.001	5.585 (3.586-8.700)	<0.001
Sarcopenia				
No	Ref			
Yes	1.780 (1.331-2.382)	<0.001	1.259 (0.921-1.721)	0.148
ONS				
No	Ref			
Yes	0.761 (0.585-0.990)	0.042	0.751 (0.573-0.985)	0.038
Muscle loss				
No	Ref			
Yes	0.902 (0.697-1.167)	0.433		
complications				
No	Ref			
Yes	1.413 (1.072-1.863)	0.014	1.055 (0.788-1.413)	0.718
NLR				
low	Ref			
high	1.399 (1.078-1.815)	0.012	0.963 (0.730-1.272)	0.791
Chemotherapy				
No	Ref			
Yes	1.204 (0.924-1.567)	0.169		

Statistically significant (P<0.05). CCI, Charlson Comorbidity Index; TNM, Tumor Node Metastasis; BMI, body mass index; ONS, oral nutritional supplements; NLR, Neutrophil to Lymphocyte Ratio.

### 3.8 Subgroup analysis after PSM

**Table 8** shows that subgroup analysis indicates short-term postoperative oral nutritional support is associated with more favorable overall survival in the following populations: hypoalbuminemia (HR = 0.698, P = 0.036), total gastrectomy (HR = 0.669, P = 0.027), TNM stage I (HR = 0.265, P = 0.009), sarcopenia (HR = 0.513, P = 0.013), and postoperative muscle loss (HR = 0.514, P = 0.001).

**Table 8:** Subgroup analysis after PSM.

Factors	Univariate analysis HR (95%CI)	P
Age		
≤ ≤65	0.706 (0.456-1.091)	0.117
> 65	0.772 (0.554-1.076)	0.126
Hypoalbuminemia		
No	0.698 (0.498-0.997)	0.036
Yes	0.856 (0.559-1.310)	0.474
Anaemia		
No	0.784 (0.537-1.143)	0.206
Yes	0.695 (0.481-1.004)	0.053
Type of resection		
distal gastrectomy	0.788 (0.531-1.169)	0.236
total gastrectomy	0.669 (0.469-0.954)	0.027
TNM		
I	0.265 (0.098-0.716)	0.009
II	1.093 (0.554-2.158)	0.798
III	0.885 (0.654-1.198)	0.430
Sarcopenia		
No	0.895 (0.651-1.203)	0.436
Yes	0.513 (0.303-0.868)	0.013
Muscle loss		
No	1.030 (0.715-1.484)	0.873
Yes	0.514 (0.341-0.775)	0.001

Statistically significant (P<0.05).

## 4 Discussion

A number of studies have demonstrated the potential of postoperative oral nutrition to mitigate muscle loss following surgeries. Kimura et al. indicated that postoperative nutritional support not only positively affects short-term weight loss after surgery but also extends its benefits up to a year later (14). Xie et al. found that the incorporation of oral nutrition post-surgery is both feasible and safe. This approach prevents body weight and BMI loss, significantly improving the patients’ quality of life (15). In the present study, the results obtained were consistent with those of the above studies, demonstrating that short-term postoperative oral nutrients were able to attenuate postoperative skeletal muscle loss in patients. Although the multifactorial analysis in the present study showed that oral nutrients did not improve the long-term prognosis of the patients, the univariate analysis and the Kaplan-Meier analysis still suggested the importance of oral nutrients for the long-term prognosis of the patients. Moreover, after propensity matching, the ONS remains valid for patient survival rates. In subgroup analyses, it was shown that in preoperatively anemia, total gastrectomized patients with low TNM stage and Sarcopenia, ONS still reduces the risk of death in this group of patients. ONS contains casein, vegetable oils, maltodextrin, minerals, vitamins, and trace elements. While meeting patients’ daily energy requirements, it increases casein intake. Deoxygenase activity rises with higher dietary casein levels, thereby enhancing muscle protein synthesis (16).

Low serum albumin levels reflect the severity of inflammation, as several studies indicate (17). Hypoalbuminemia correlates with adverse outcomes such as reduced quality of life, shortened lifespan (18–20), postoperative anastomotic leakage, and weight loss (21, 22). Persistent inflammation can drive a negative nitrogen balance, leading to skeletal muscle loss post-surgery. Improvements in albumin levels and increased nutritional intake indicate a positive impact on overall nutritional status. Albumin is considered a common marker for assessing nutritional status in cancer patients. Studies have found that serum albumin levels are influenced by various physiological factors, such as age and blood loss. Although multivariate analysis in this study did not reveal a significant impact of hypoalbuminemia, propensity score-matched analysis demonstrated that preoperative hypoalbuminemia still affected patient outcomes. Preoperative hypoalbuminemia often leads to persistent negative nitrogen balance postoperatively. This exacerbates the reduction in skeletal muscle mass after surgery, ultimately affecting long-term patient outcomes. Therefore, timely correction of hypoalbuminemia is essential for individuals with low albumin levels.

CCI is the most commonly used for assessing comorbidities, also predicts the onset of sarcopenia (23). Multiple studies demonstrate that higher CCI scores correlate with a greater likelihood of muscle loss after surgery (24). The coexistence of multiple diseases accelerates nutrient consumption and raises the risk of muscle loss. In our study, individuals with high CCI scores were more prone to muscle loss postoperatively, though no impact on long-term prognosis was observed.

Sarcopenia reflects the patient’s muscle condition before surgery. Continuous negative nitrogen balance during inflammatory periods post-surgery may lead to muscle mass reduction (25), Studies have shown that sarcopenia predicts postoperative mortality in cancer patients, which is consistent with our findings (26). Among patients included in this study, those with sarcopenia were more prone to postoperative muscle loss, which adversely affected their prognosis. Oral nutritional supplements (ONS) attenuated postoperative muscle loss and enhanced long-term outcomes. Sarcopenia leads to severe declines in strength and mobility, potentially prolonging bed rest and reducing quality of life. The interaction between sarcopenia and disease may cause more pronounced physical decline and accelerate tumor progression, further shortening overall survival (OS) in patients. This study demonstrates that postoperative nutritional intervention is crucial for individuals with sarcopenia.

TNM stage has a significant impact on patient prognosis; the higher the stage, the worse the survival rate. Consistent with this, our results suggest that the higher the stage of TNM staging, the higher the risk of death and the decline in physical condition, mental health and overall quality of life of patients with advanced gastric cancer (27). Short-term postoperative nutritional interventions are not sufficient to ameliorate long-term nutritional deficiencies in patients, further contributing to shorter OS time in patients with high TNM staging. However, for patients with early-stage gastric cancer, who generally have better physical condition, mental health, and overall quality of life compared to those with advanced cancer, nutritional intervention is essential.

Anemia has become a prevalent post-gastrectomy complication, and there is a potential relationship between post-gastrectomy anemia, nutritional deficiencies, and poor prognostic outcomes, usually attributed to iron, vitamin B12, or folate deficiencies, either alone or synergistically (28). These deficiencies are often caused by malabsorption, reduced dietary intake, or chronic gastric mucosal bleeding, and in patients with anemia, postoperative nutritional supplements can provide the nutrients patients need and reduce the risk of death.

Postoperative weight loss is related to the patient’s tumor (29, 30); tumor progression leads to weight loss, and as gastric cancer progresses to advanced stages, weight loss may be due to dysphagia, painful swallowing, anorexia (31), or cancer cachexia (32), and for the patient, preoperative BMI correlates with nutritional status, and a higher BMI within the normal range mitigates against further postoperative skeletal muscle loss. Although muscle loss was not significantly associated with patient overall survival in this study, this may be due to insufficient sample size.

Total gastrectomy was associated with more severe biochemical and nutritional alterations compared to subtotal procedures. Total gastrectomy significantly exacerbates early postoperative metabolic and nutritional derangements (33). Persistent negative nitrogen balance can exacerbate loss of skeletal muscle and reduce patients’ quality of life, and the interaction between nutritional deficiencies and disease further contributes to shorter OS in patients. Our subgroup analysis showed that ONS remained significant in the total gastrectomy population. This emphasizes the importance of postoperative oral nutrition to reduce the risk of mortality in patients. However, this study indicates that patients undergoing distal gastrectomy experience greater muscle loss postoperatively (14).Previous reports suggest that oral nutritional supplements can improve postoperative weight loss in total gastrectomy patients but do not alleviate weight loss in distal gastrectomy patients. This discrepancy is speculated to stem from the nutritional interventions administered to total gastrectomy patients postoperatively.

Chemotherapy is a risk factor that affects skeletal muscle loss in cancer patients after surgery. Studies have shown that, compared with healthy individuals, patients undergoing chemotherapy exhibit significant impairment in muscle strength, with notable differences from healthy populations (34). There are four main causes of muscle loss during chemotherapy: (I) reduced vitamin D leading to impaired food intake; (II) decreased omega-3 fatty acids and protein; (III) reduced physical activity secondary to fatigue; (IV) direct effects of chemotherapy or targeted drugs on muscles; (V) malabsorption secondary to mucositis or treatment-related pancreatic insufficiency (35). Therefore, chemotherapy patients often face the risk of postoperative skeletal muscle loss.

Nonetheless, this study possesses limitations. Firstly, its retrospective nature and confinement to a single center may affect the generalizability of findings. Secondly, there is still a lack of research on specific postoperative chemotherapy regimens, blood glucose levels, and other relevant indicators that affect postoperative skeletal muscle mass loss and survival. Therefore, further exploration is needed.

Postoperative ONS becomes a strategy to mitigate the loss of skeletal muscle mass in patients with gastric cancer, especially important in patients with multiple comorbidities and abnormal biochemical markers. more clinical attention should be paid to postoperative nutritional interventions for patients.

## Data Availability

The raw data supporting the conclusions of this article will be made available by the authors, without undue reservation.
