# Supplementary material for: Effect of short-term oral nutrients after hospital discharge on postoperative muscle loss and survival in gastric cancer patients
Source: Front Oncol. 2026 Jan 6;15:1697609. doi: 10.3389/fonc.2025.1697609 (PMC12815837; doi:10.3389/fonc.2025.1697609)
Supplement: Supplementary file 1 [file DataSheet1.pdf]

The Kaplan–Meier curve of subgroups.

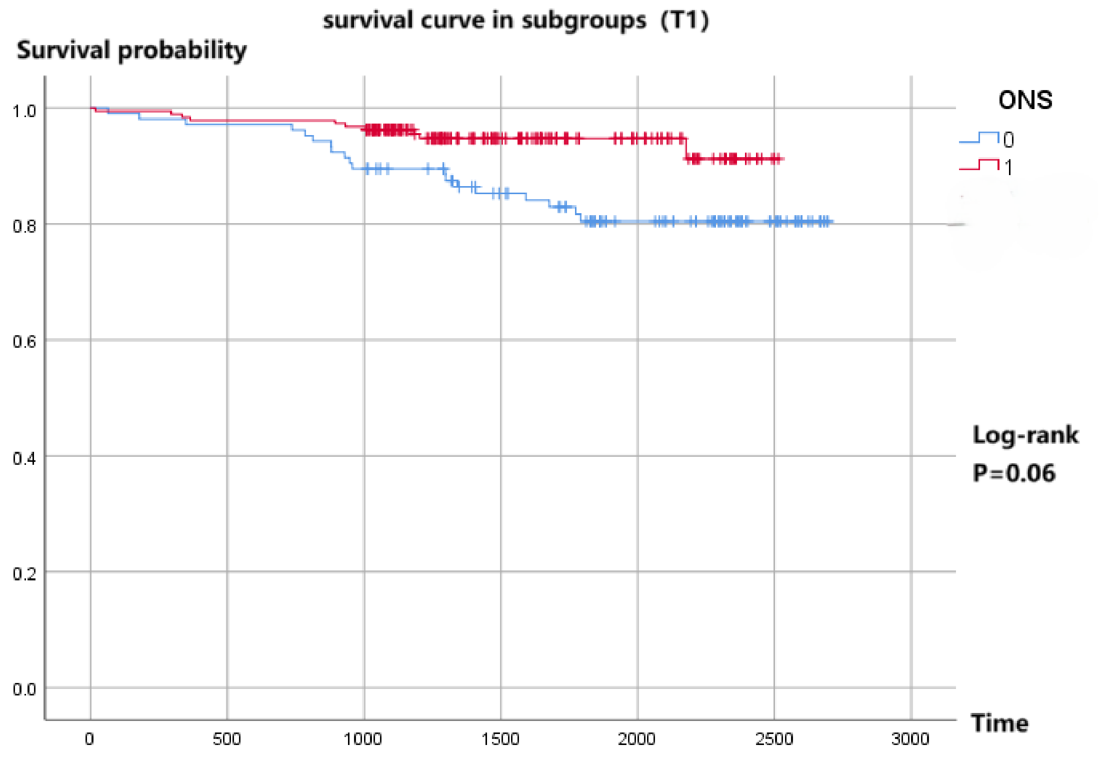

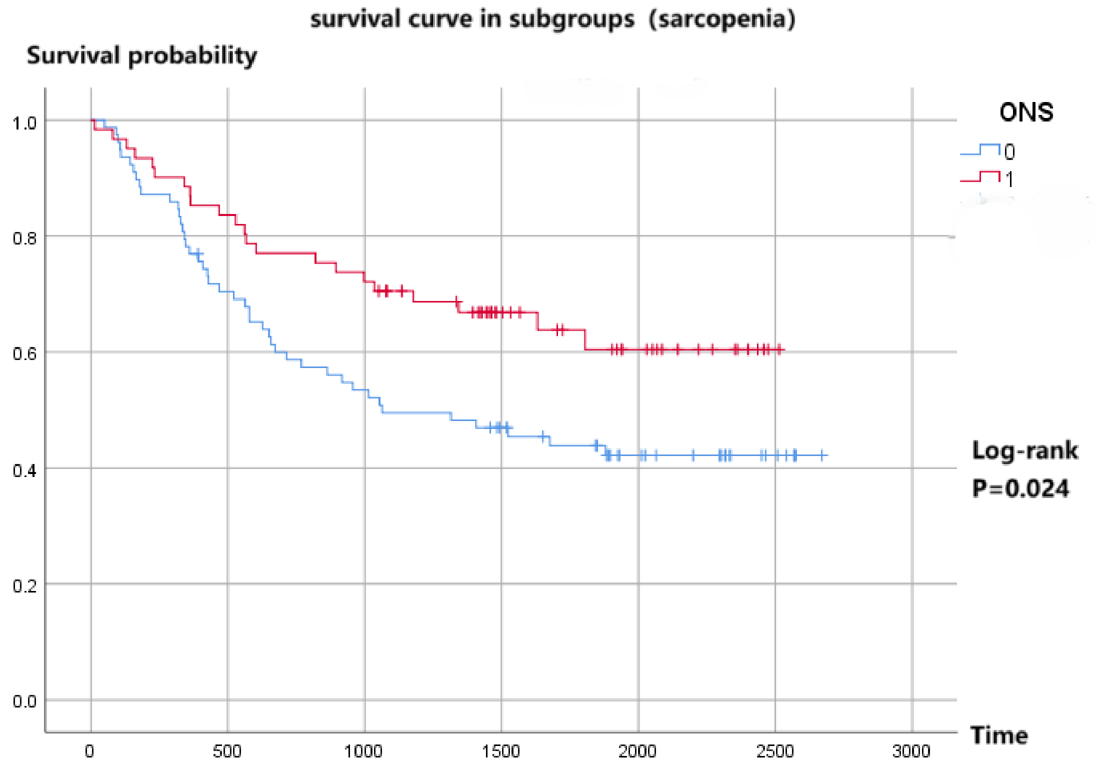

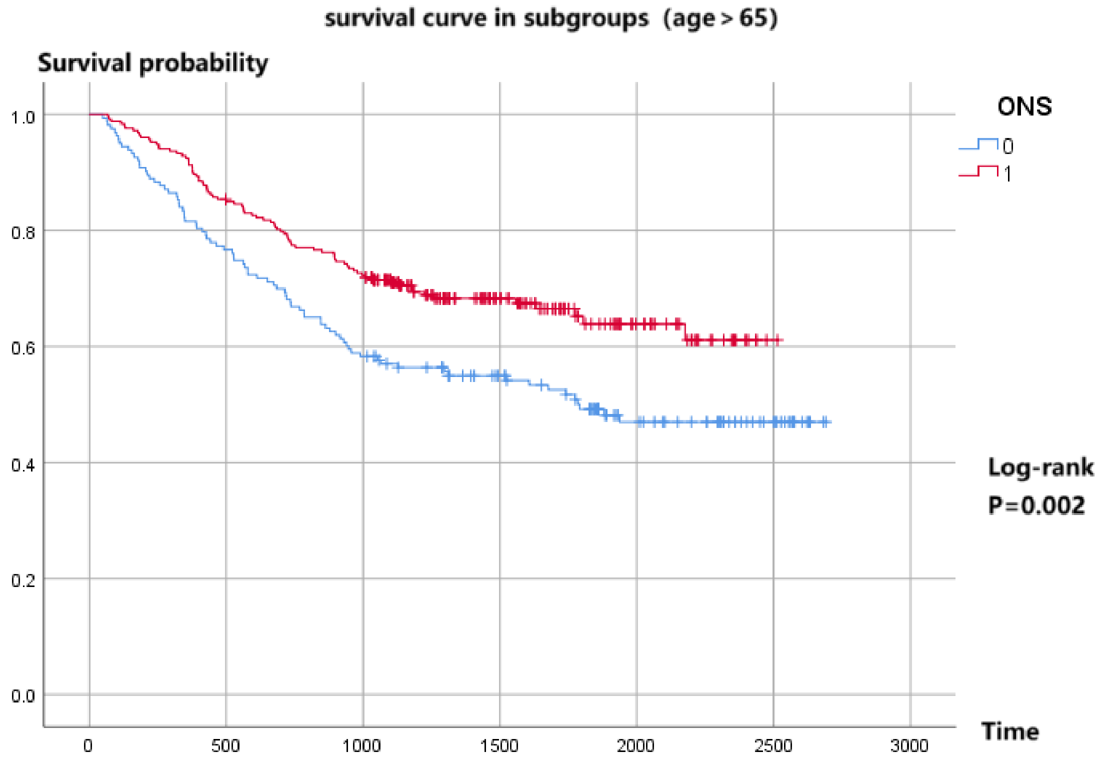

survival curve in subgroups (age＞65)
Survival probability
1.0
0.8
0.6
0.4
0.2
0.0
0
500
1000
1500
2000
2500
3000
Time
ONS
0
1
Log-rank
P=0.002

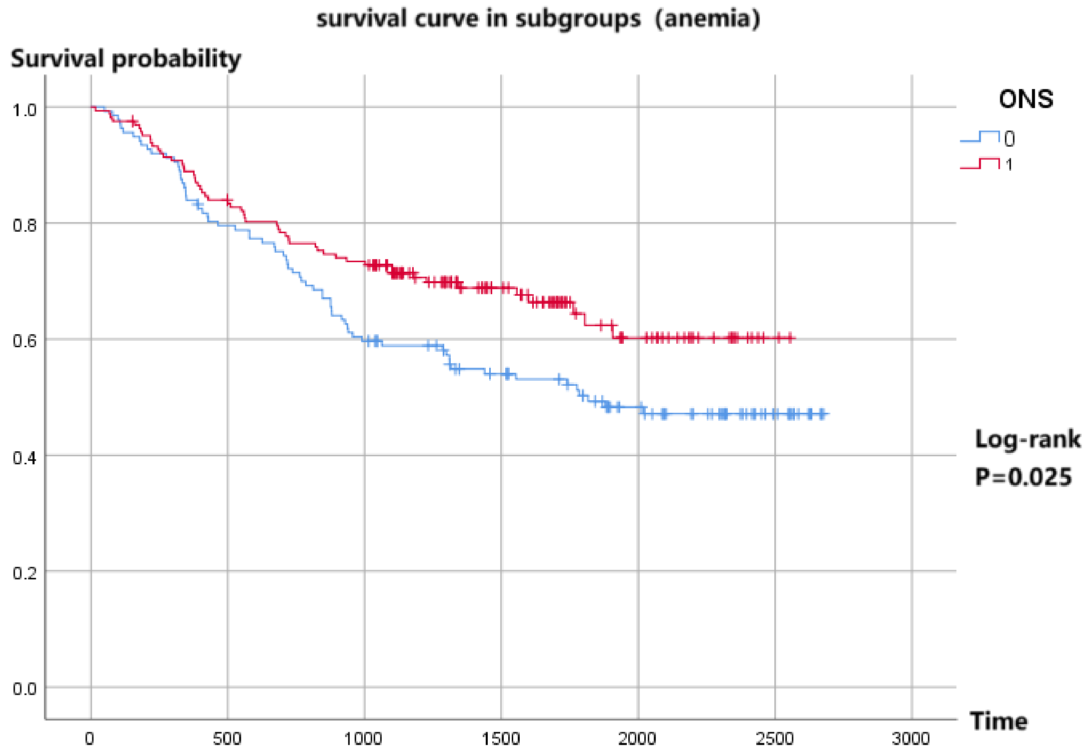

survival curve in subgroups (anemia)
Survival probability
1.0
0.8
0.6
0.4
0.2
0.0
0
500
1000
1500
2000
2500
3000
Time
ONS
0
1
Log-rank
P=0.025

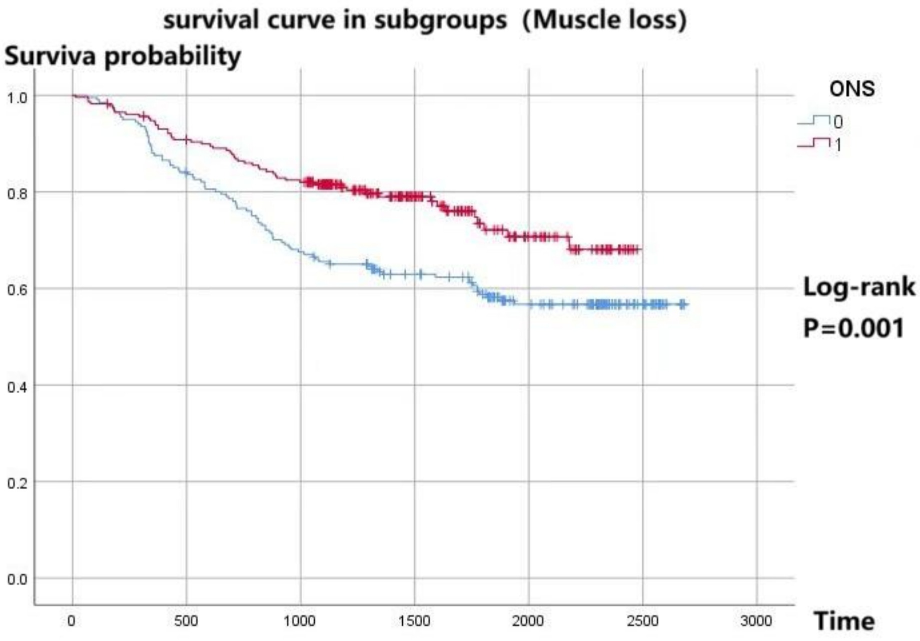
survival curve in subgroups（Muscle loss）
Surviva probability
1.0
0.8
0.6
0.4
0.2
0.0
0
500
1000
1500
2000
2500
3000
Time
ONS
0
1
Log-rank
P=0.001

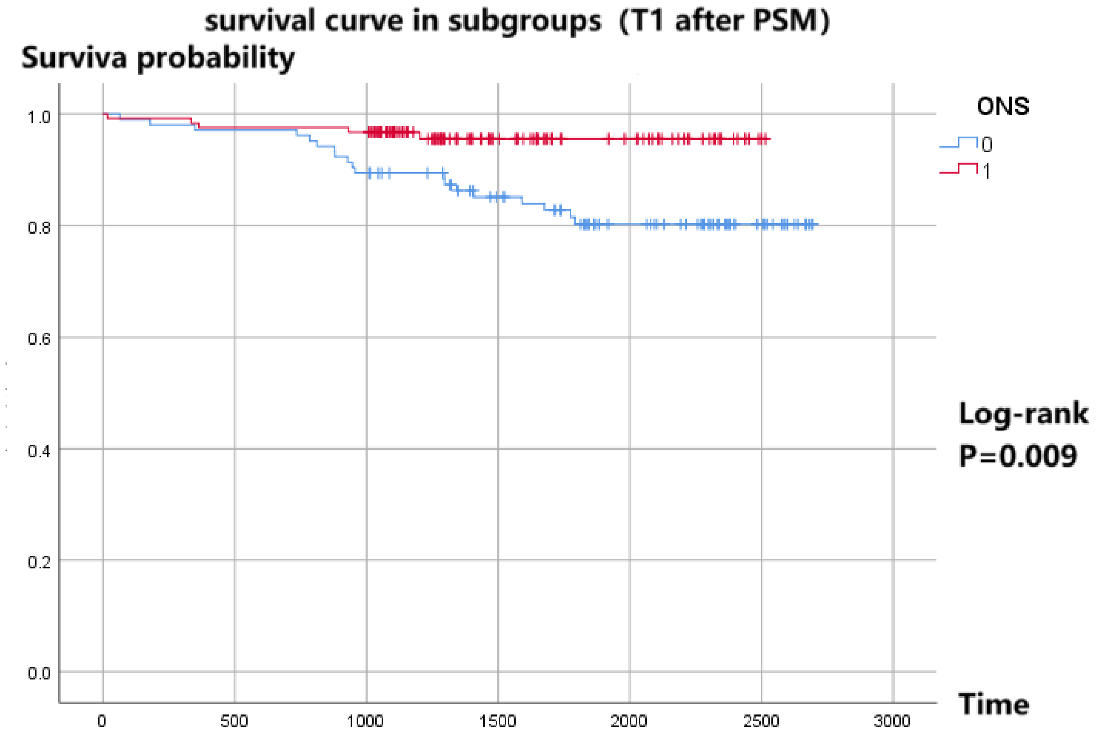
survival curve in subgroups（T1 after PSM）
Surviva probability
1.0
0.8
0.6
0.4
0.2
0.0
0
500
1000
1500
2000
2500
3000
Time
ONS
0
1
Log-rank
P=0.009

survival curve in subgroups（Sarcopenia after PSM）

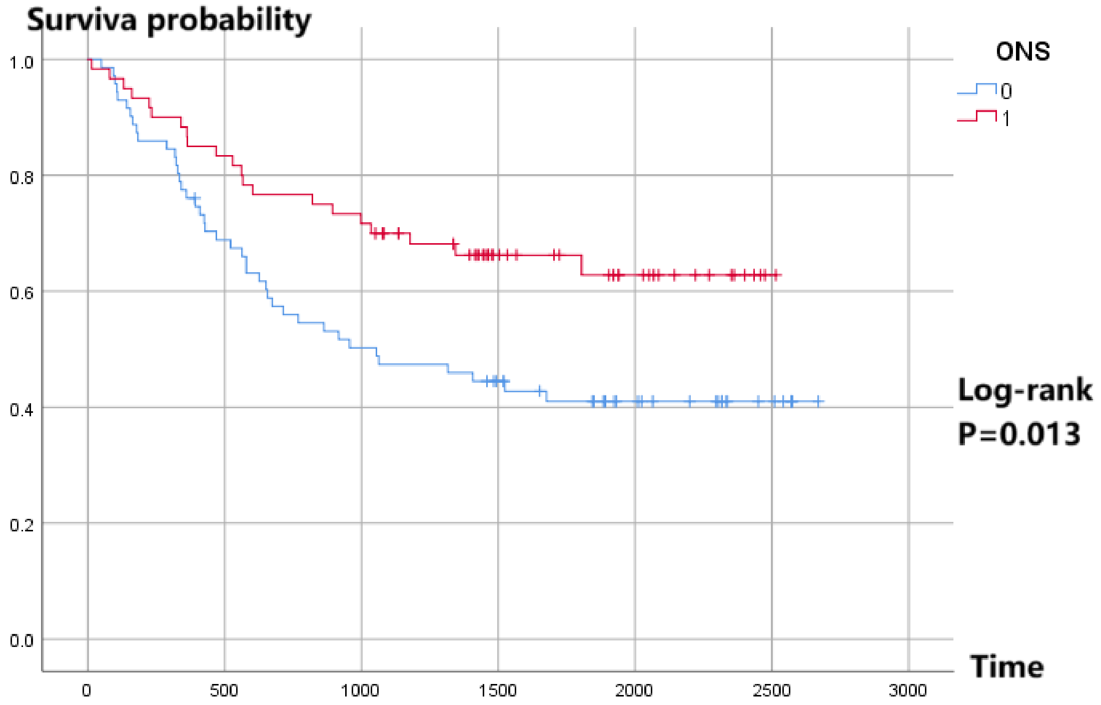

survival curve in subgroups（TG after PSM）

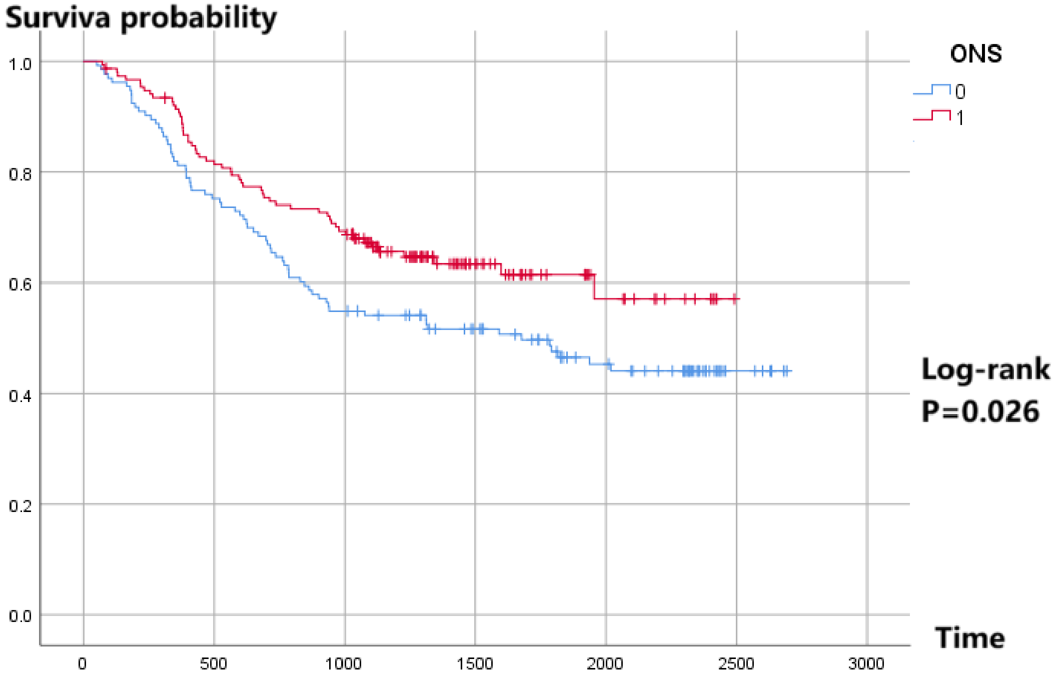

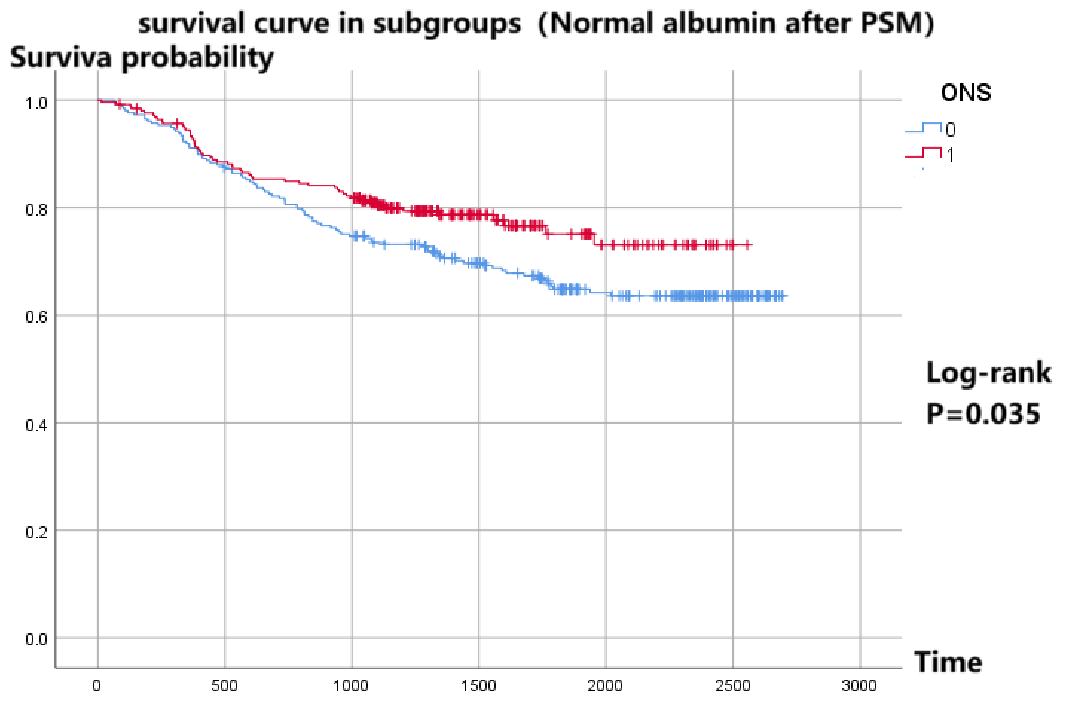
survival curve in subgroups（Normal albumin after PSM)
Surviva probability
1.0
0.8
0.6
0.4
0.2
0.0
0
500
1000
1500
2000
2500
3000
Time
ONS
0
1
Log-rank
P=0.035

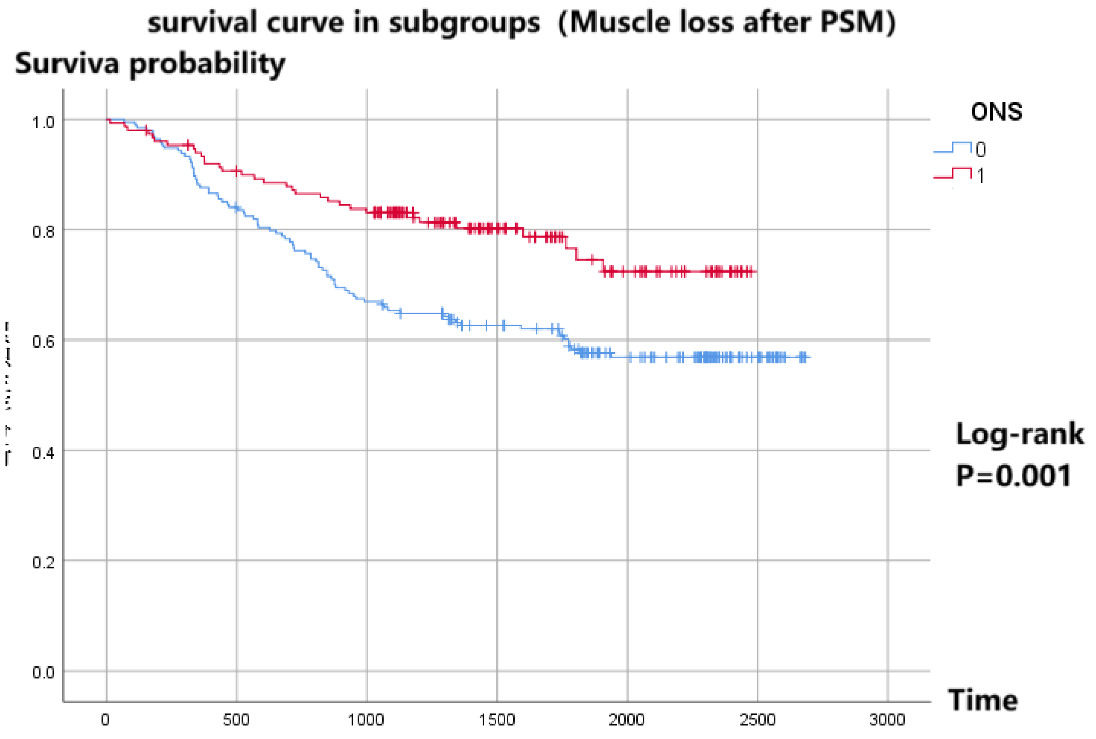
survival curve in subgroups（Muscle loss after PSM)
Surviva probability
1.0
0.8
0.6
0.4
0.2
0.0
0
500
1000
1500
2000
2500
3000
Time
ONS
0
1
Log-rank
P=0.001
