# Supplementary material for: Effect of short-term oral nutrients after hospital discharge on postoperative muscle loss and survival in gastric cancer patients
Source: Front Oncol. 2026 Jan 6;15:1697609. doi: 10.3389/fonc.2025.1697609 (PMC12815837; doi:10.3389/fonc.2025.1697609)
Supplement: Supplementary file 2 [file DataSheet2.pdf]

1.Dietary tables for two groups

	Diet
Normal diet	Normal diet
Normal diet+ONS	Each patient follows a normal diet and additionally takes 500ml of nutritional supplements daily for 1 month.<br>1.Normal diet+Enteral Nutritional Suspension: (Each 500ml contains energy 500kcal, protein 20.0g, carbohydrates 61.5g, fat 19.45g.)<br>2.Normal diet+Intacted Protein Enteral Nutrition Powder: (Every 100 mL of liquid is made by mixing 21.5 g of powder. Each 500ml contains 496.65 kcal of energy, 19.89g of protein, 60.63g of carbohydrates, and 19.57g of fat.).

2.Pie chart of muscle loss, hypoalbuminemia, and tumor risk with and without ONS diet

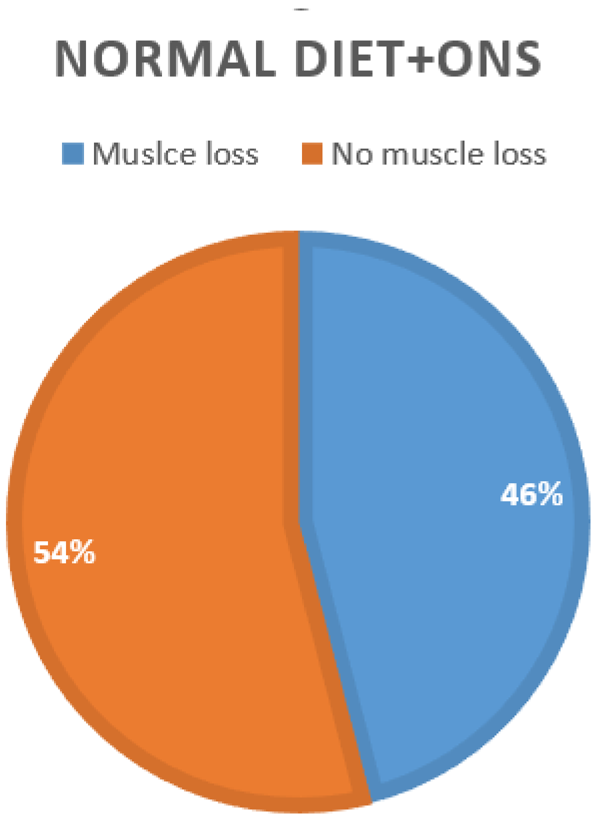

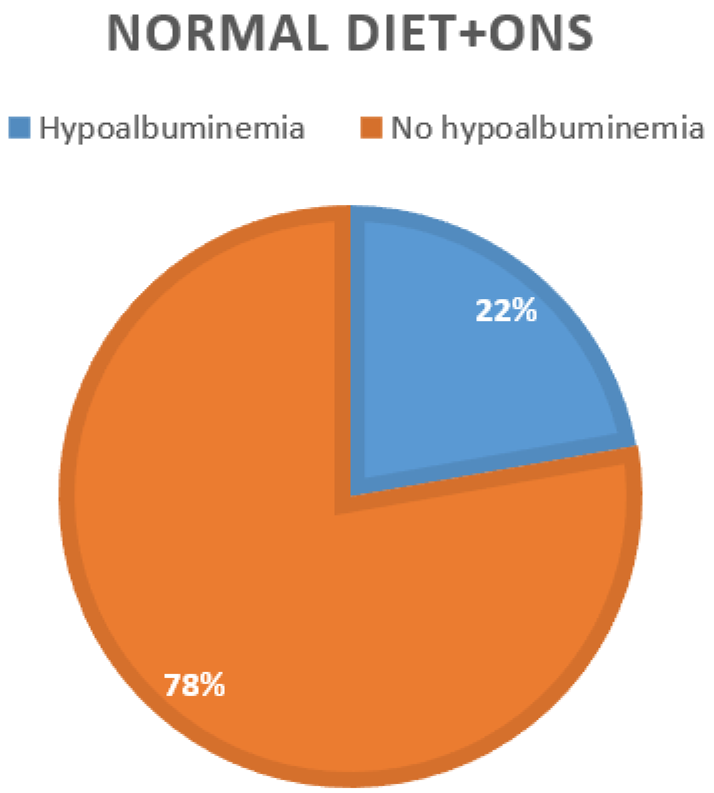
NORMAL DIET+ONS
Hypoalbuminemia
No hypoalbuminemia
22%
78%

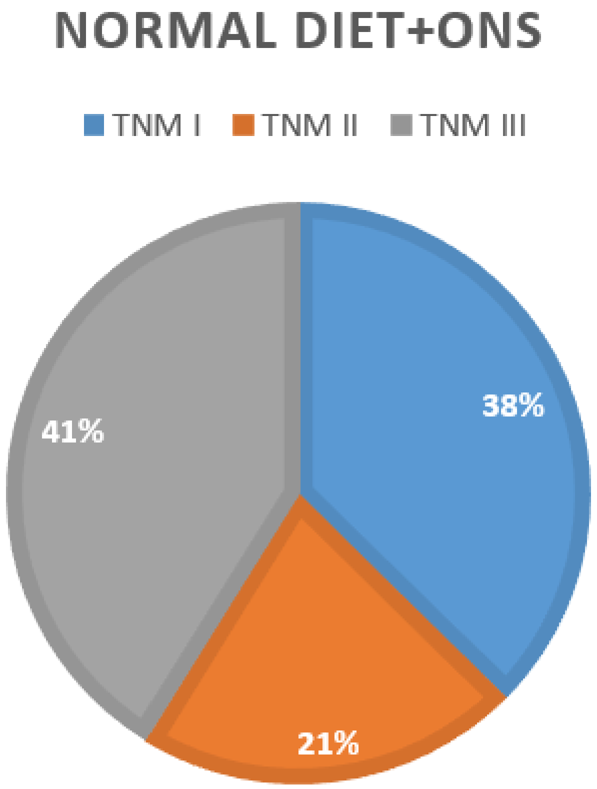
NORMAL DIET+ONS
TNM I
TNM II
TNM III
38%
21%
41%

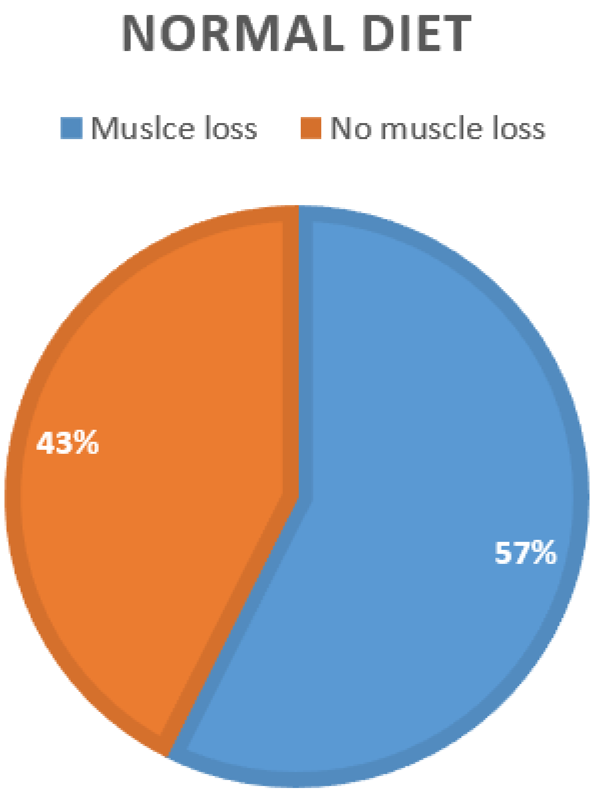
NORMAL DIET
Muslce loss
No muscle loss
43%
57%

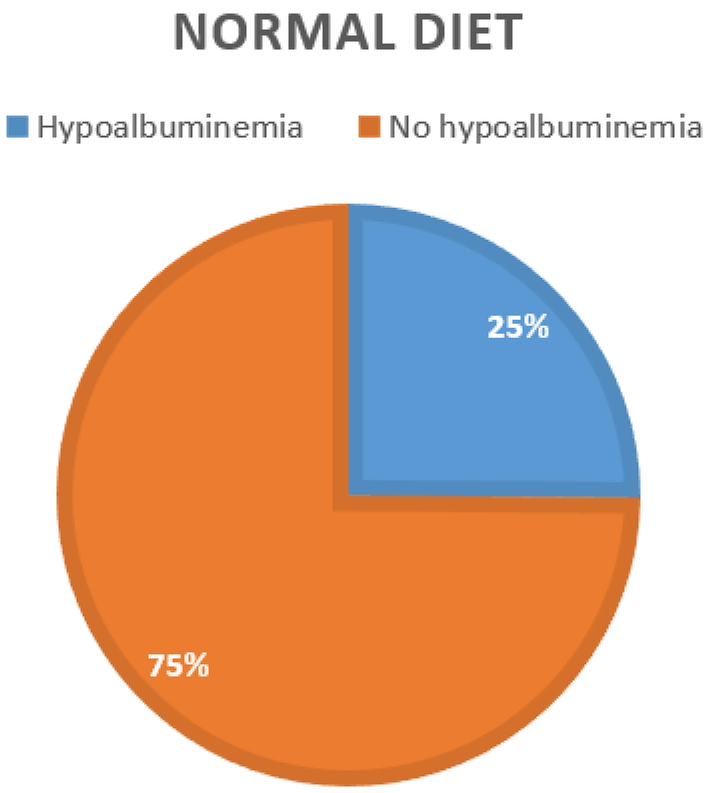
NORMAL DIET
Hypoalbuminemia
No hypoalbuminemia
25%
75%

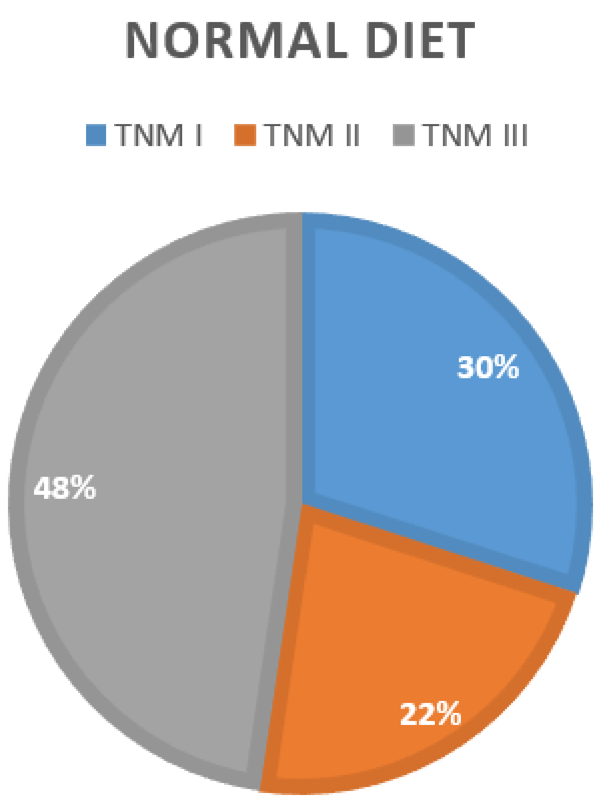
NORMAL DIET
TNM I
TNM II
TNM III
30%
22%
48%
